# Anatomical and physiological contributions of nasal turbinate vessels and lymphatics to the pathogenesis of nasal congestion in recurrent headaches: a pilot study

**DOI:** 10.3389/fpain.2025.1521500

**Published:** 2025-02-05

**Authors:** Jacob M. Chmielecki, Aishwarya Vemula, Joyce G. Schwartz, Jonathan A. Gelfond, William T. Phillips

**Affiliations:** ^1^ Department of Radiology, UT Health, San Antonio, TX, United States; ^2^ Department of Pathology, Methodist Hospital, San Antonio, TX, United States; ^3^ Population Health Sciences, UT Health, San Antonio, TX, United States

**Keywords:** glymphatics, headaches, migraines, nasal turbinate, topiramate, whole-body blood pool scintigraphy

## Abstract

**Introduction:**

The aim of this study was to determine if specific anatomical changes were present in patients with recurrent headaches including patients with chronic migraines, chronic tension-type headaches, and daily persistent headaches. A retrospective study of 200 patients was performed to evaluate the potential of measuring the amount of nasal blood pool activity (nasal congestion) as a predictive marker for recurrent headaches.

**Methods:**

A cohort analysis was performed involving patients who had been referred to the Nuclear Medicine Clinic over a 3-year period for whole-body blood pool scans. The scans were evaluated by region of interest (ROI) analysis of nasal and heart max pixel count ratios (NHMRs) to determine an association between nasal blood pooling activity and recurrent headaches at the time of the initial scan and in follow-up evaluations over a period of 3–6 years.

**Results:**

Significantly greater NHMRs were observed in 122 patients with chronic headaches at the time of referral for their initial whole-body blood pool scan when compared with those patients without recurrent headaches (*p* = 0.004; OR 10.5; 95% CI 2.22–56.7). An additional 15 patients, for a total of 137, developed recurrent headaches following their initial scan and before their follow-up evaluation. NHMRs were also significantly increased in the additional patients when compared to those without recurrent headaches (*p* = 0.004; OR 12.3; 95% CI 2.34–75.5).

**Conclusion:**

Patients with recurrent headaches have significantly increased nasal activity as observed on ^99m^Tc-MDP whole-body blood pool scans, supporting the hypothesis that nasal lymphatic dysfunction plays a role in the etiology of recurrent headaches. This research highlights a novel diagnostic use of the whole-body blood pool scan for the assessment of nasal turbinate vasodilation as well as a possible new target for the treatment of recurrent headaches.

## 1 Introduction

Fifty-six percent of adults in the United States get a headache at least once a month. Twenty percent have at least one headache a week, including five percent who suffer from a headache almost daily (1). The International Headache Society defines chronic headaches as, “15 or more headache episodes per month for at least three months (2).” It includes subtypes such as chronic migraine, tension-type headache, medication overuse headache, hemicrania continua, and new daily persistent headache, as per ICHD-3 guidelines (3).

For this study the authors categorized patients as having recurrent headaches if this diagnosis was documented in their current medical chart. The information regarding headaches was placed in the patients’ charts by their primary care physician, their rheumatologist, or by another physician the patients had seen. Although the information in some patients’ charts specifically described chronic migraines, chronic tension-type headaches, or daily persistent headaches, frequently the type of headache, the frequency, severity and duration were omitted. Because of these issues, the term “recurrent” headache will be used in this article as opposed to “chronic primary” headache.

Migraines remain a significant health issue, leading to approximately 4.3 million physician office visits and 4 million visits to the emergency department in 2016 alone (4). The global burden of these conditions is substantial, with migraines ranking highly among causes of disability, especially among young women (5). According to the latest Global Burden of Disease study, headache disorders rank as the second leading cause of disability among individuals aged 10–24 years and the fifth most common in the 25–49-year age group (6). Recent studies have also reported that patients with both migraine and non-migraine headaches have a 4–6-fold increase in suicide attempts (7).

Recent articles have proposed that recurrent headaches, specifically migraine headaches, may be characterized by two opposing processes: lack of habituation (a reduction of inhibitory response to repeated sensory stimuli) and sensitization (an augmentation of response to repeated sensory stimuli) (8, 9). It is also proposed that musculoskeletal disorders may act as a trigger or risk factor for headache chronification. Deodato et al. highlighted the bidirectional relationship between primary headaches and musculoskeletal disorders, especially in the cranio-cervical areas such as postural alterations and musculoskeletal dysfunctions (9).

Anatomic variants like septal deviations, concha bullosa, and variations in uncinate and ethmoid structures can obstruct sinus outflow tracts and lead to headaches, suggesting an anatomical link between nasal pathology and headache syndromes (10). Recent research has begun to explore the anatomical and physiological contributions of nasal turbinates to the pathogenesis of recurrent headaches (11). The nasal turbinates, which are crucial for air humidification, warming, and filtration, may play a role in headache onset when inflamed or hypertrophied (12, 13). Studies over the last two decades have also shown that the nasal turbinate lymphatics are important sites of cerebrospinal fluid (CSF) clearance through the cribriform plate (14–18). An excellent review of the nasal lymphatic route of CSF outflow along the olfactory nerves through the cribriform plate and its potential contribution to neurodegenerative disease has been recently published by Chae et al. (14).

Due to their increasing prevalence and complex etiology, recurrent headaches have emerged as a focal point of research interest. In a prior study, we observed that patients with metabolic syndrome components, including elevated body mass index (BMI), had significantly increased nasal blood pool activity (19). We also observed that these same patients with increased nasal blood pool activity were frequently noted to have the diagnosis of recurrent headaches and migraines. The objective of this study was to determine if the clinically observed increased nasal blood pool activity observed on whole-body scans in patients with recurrent headaches could be verified in a large-scale retrospective study using quantitative region of interest analysis.

The possibility that patients with recurrent headaches have increased nasal blood pool activity, likely due to nasal turbinate vasodilation, could have important implications for a variety of medical conditions such as chronic sinus congestion, nasal infections, and other medical conditions affecting the nasal turbinates. Although there is no current panacea for recurrent headaches, through this study, we aspire to contribute meaningful discourse surrounding the cause of headaches and the mechanism of topiramate, an antiseizure medication also used to prevent the occurrence of migraine headaches. We suggest that excessive nasal turbinate vasodilation may be a novel therapeutic target for the treatment of headaches and migraines.

After conducting a thorough review of existing literature, the authors contend that this article marks the initial recorded instance of the connection between increased nasal blood pool activity in individuals diagnosed with recurrent headaches.

## 2 Materials and methods

### 2.1 Study design and population

The authors conducted a retrospective analysis involving 200 patients referred from the rheumatology clinic at University Hospital in San Antonio for whole-body blood pool imaging. Each patient was imaged only once. The patients’ medical charts were reviewed up to 6 years following their blood pool imaging study to ascertain if headaches had developed. Although the majority of patients studied had documentation in their medical records of recurrent headaches, the primary or chief complaint of these individuals was related to their joint damage, therefore they were being seen in, and referred from, a rheumatology clinic as opposed to a neurology clinic.

The Institutional Review Board granted approval for a retrospective evaluation of this study (HSC20200389E). A retrospective data collection process was carried out on the same 200 patients from a previous study performed at the University Hospital Nuclear Medicine Clinic. Patient anonymity was diligently maintained throughout the article, rendering patient consent unnecessary. The review encompassed the period from May 1, 2017, to May 1, 2023, with individuals under 18 years of age and over 80 years of age excluded from the study. The patients in the rheumatology clinics had undergone whole-body blood pool imaging as part of their assessment for joint and other musculoskeletal-related inflammation.

Imaging was performed to detect sites of inflammation as the first part of a standard whole-body *bone scan* performed 3 h post-injection (20, 21). The whole-body *blood pool scan* is performed within the first few minutes after injection of 25 mCi of the bone agent, technetium-99m-methylene diphosphonate (^99m^Tc-MDP), while it remains in the blood pool prior to its deposition in the bone. Blood pool imaging was analyzed for a pattern of increased uptake in the nasal turbinate region, which was quantified by the ratio between max pixel count in the nasal turbinate region vs. the heart (see Section 2.3: imaging acquisition and analysis methodology).

Data for this research was collected and managed using REDCap electronic data capture tools hosted by UT Health San Antonio (22, 23). REDCap (Research Electronic Data Capture) is a secure web-based software platform designed to support data capture for research studies, providing: (1) an intuitive interface for validated data capture, (2) audit trails for tracking data manipulation and export procedures, (3) automated export procedures for seamless data downloads to common statistical packages, and (4) procedures for data integration and interoperability with external sources. Previously collected data for each patient included the following information: diagnosis of diabetes, impaired glucose tolerance, hypertension, sleep apnea, hyperlipidemia, whether the patient was taking medication for diabetes, hypertension, or hyperlipidemia, BMI, an average of two recent fasting glucose levels, two recent blood pressures, hemoglobin A1c, triglycerides, HDL, LDL, and total cholesterol. The 200 patients were representative of the San Antonio population, with over half having Hispanic surnames.

For the current research, additional data was collected regarding a history of recurrent headaches. The authors listed patients as having recurrent headaches if this diagnosis was documented in their current medical chart. Those patients with recurrent headaches included those with chronic migraines, chronic tension-type headaches, and daily persistent headaches. Frequently the specific type of recurrent headache was not described in the patients’ medical records. Variables included whether the patient suffered from recurrent headaches at the time of the initial blood pool scan (scans were taken 2017–2020), if they began suffering from recurrent headaches 3–6 years later at the time of data collection for this study (2023), what subtype of headaches they were suffering from if they did have headaches, and if they were taking prescription medication at the time of the initial scan, the name of the medication [which included topiramate, sumatriptan, amitriptyline, or a butalbital/acetaminophen/caffeine combination (Fioricet)].

### 2.2 Statistics

The primary hypothesis was that recurrent headaches are positively associated with elevated nose-to-heart max ratios (NHMR). All 200 patients who had undergone whole-body blood pool imaging were included in the study. We used Mann–Whitney *U* tests to determine if the presence of recurrent headaches at the time of the initial scan and at the time of follow-up correlated with elevated NHMRs. We then used multivariable logistic regression to assess the predictive value of the following variables on patients’ burden of headaches: gender, diabetes, hypertension, sleep apnea, hyperlipidemia, antihypertensive medication, anti-lipid medication, antihyperglycemic medication, and NHMR. We selected these variables because there are known associations between metabolic syndrome and headaches and because we wanted to verify that nose-to-heart max ratios made a unique contribution to headaches, independent of metabolic syndrome. We did not include BMI, hemoglobin A1c, blood pressure, blood glucose levels, high-density lipoprotein (HDL), low-density lipoprotein (LDL), or total cholesterol as independent variables for the statistical analysis because there were patients for whom this data was unavailable, and we did not want to omit them from the study. Dependent variables in the analysis were whether the patient suffered from recurrent headaches at the time of the initial scan, and whether the patient developed recurrent headaches 3–6 years later at the time of data collection (2023). For all the predictor variables, we used the data that had been collected at the time the scans were performed. We collected both information obtained at the time of the initial scan and updated information 3–6 years after the initial scan for information regarding recurrent headaches because we wanted to assess whether the independent variables were already contributing to headaches at the time that they were recorded, as well as whether they contributed to the development of headaches at a later time.

We also performed an analysis to determine if any of the drugs were associated with a reduced NHMR. We used a multivariable regression analysis to assess which of the following variables contributed to predicting a patient's NHMR: whether the patients had recurrent headaches at the time of the initial scan, the number of medications (if any) they were taking for headaches at the time of the initial scan, whether they had sleep apnea, and whether they were specifically taking any of the following four medications for headaches: topiramate, amitriptyline, a combination of acetaminophen, butalbital, and caffeine (Fioricet), or sumatriptan. We also performed a *post hoc* Mann–Whitney *U* test to compare NHMR in patients with recurrent headaches who were taking topiramate to the rest of the patients with recurrent headaches.

Statistical significance was set at a two-sided level of *p* < 0.05.

### 2.3 Imaging acquisition and analysis methodology

Each patient received an injection of 20–25 mCi of the bone avid radiopharmaceutical, technetium-99 m-methylene diphosphonate (^99m^Tc-MDP). Whole-body blood pool images were obtained beginning at 2–3 min following injection of ^99m^Tc-MDP, taking 6–7 min to complete the scan from head to feet. The imaging was performed using a dual-headed gamma camera (GE Infinia Hawkeye 4 dual-head SPECT/CT camera) equipped with low-energy, high-resolution collimators. The energy window was set at 140 keV, with a 20% window moving at a rate of 36 cm per minute.

**Figure 1:**
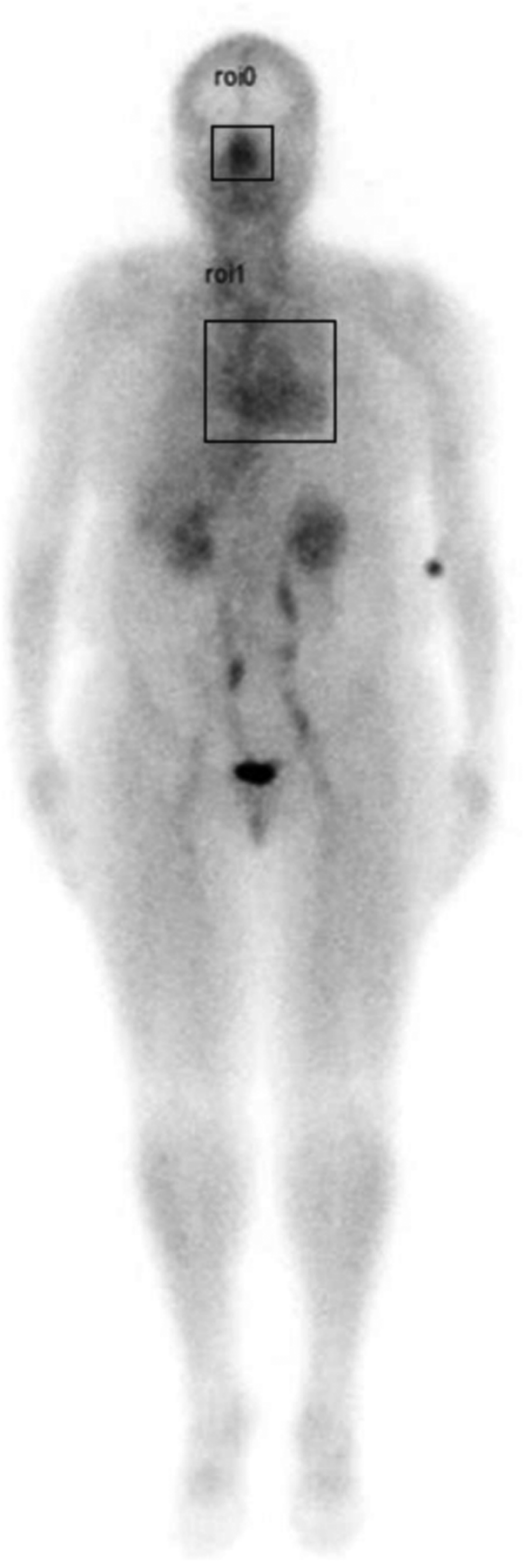
Example of whole-body blood pool image showing region of interest (ROI) areas (square boxes) over the nasal turbinate region and the heart.

Region of interest (ROI) analysis of the whole-body blood pool images was performed by drawing ROI boxes over the nasal turbinate region and the heart region, then comparing the max pixel nasal counts to the max pixel heart counts to determine a nose-to-heart max ratio (NHMR) as shown in Figure 1 using the methodology described previously (19).

## 3 Results

The 200 subjects in the study had a median BMI of 32 and 88% were female. There was no selection bias other than the fact that more women presented to the rheumatology clinic compared to men (rheumatoid arthritis is 3 times as prevalent in women than in men), and these patients were referred for whole-body nuclear medicine scans. The images were viewed in the chronological order that the scans were performed. In this population, there were substantial frequencies of diabetes (29%), hypertension (53%), hyperlipidemia (47%), and sleep apnea (26%) as shown in Table 1. Table 1 also provides information on the number of patients taking one or more medications (1+) to treat these conditions.

**Table 1:** Demographic and clinical profile of study participants at the time of initial whole-body blood pool scan.

Characteristic	*n* = 200	Missing
Female	177 (88%)	
Male	23 (12%)	
Headaches at initial scan	122 (61%)	
Diabetes	57 (29%)	
Sleep apnea	51 (26%)	
Hyperlipidemia	93 (47%)	
Hypertension	106 (53%)	
Number of anti-hypertensive medications (1+)	93 (47%)	10
Number of anti-lipid medications (1+)	69 (35%)	6
Number of anti-hyperglycemic medications (1+)	52 (26%)	7
	*M* (interquartile range)	
Age	50 (42, 58)	
BMI	32 (26, 37)	7
Average blood glucose	96 (89, 116)	3
Hemoglobin A1c	5.70 (5.40, 6.40)	29
Nose-to-heart max ratio	0.89 (0.74, 1.01)	
Average systolic BP	125 (115,135)	10

*M*, median; (1+), one or more medications; BMI, body mass index; BP, blood pressure.

**Figure 2:**
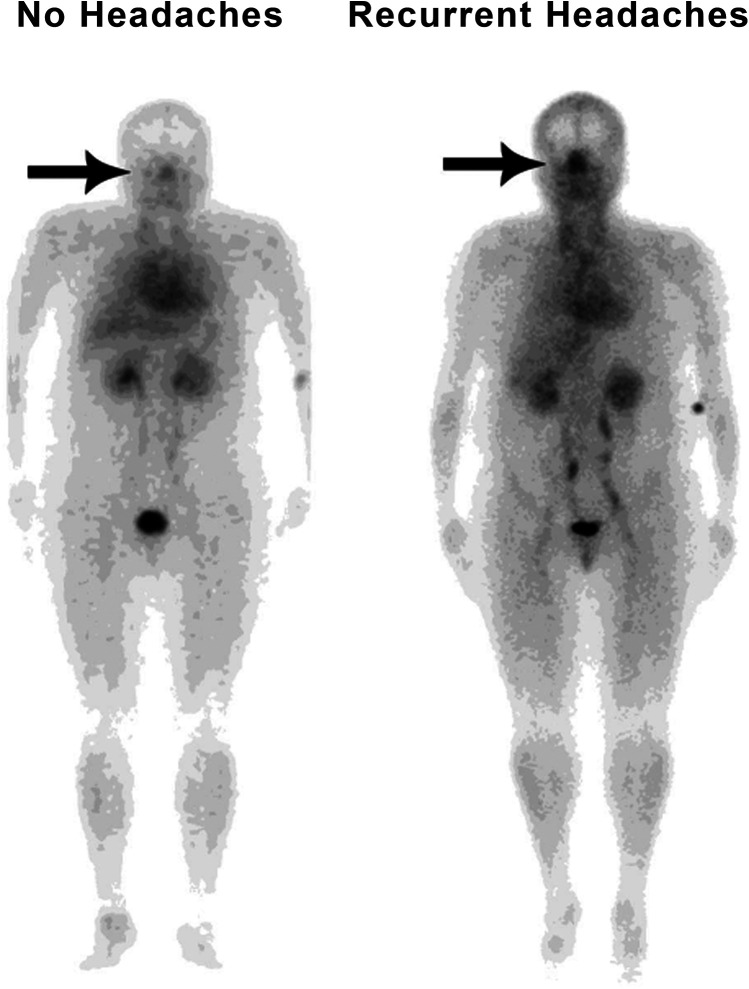
Typical whole-body blood pool image comparing a patient without headaches (with minimal nasal congestion) to a patient with recurrent headaches (markedly increased nasal congestion).

Of the 200 patients, 122 had been suffering from recurrent headaches *at the time of the initial scan*, and 78 had not. The median NHMR among the 122 suffering from headaches was 0.920 compared to 0.818 among the others (*p* = 0.004) (Table 2). Of the 122 patients with recurrent headaches, 63 (51.6%) were diagnosed with unspecified recurrent headaches without an identified subtype, 57 (46.7%) had migraines, 4 (3.3%) had tension headaches, and 1 had cluster headaches (0.8%). Two patients, already accounted for by the previous figures, experienced multiple subtypes, with one experiencing migraines and tension headaches, and the other experiencing migraines, tension headaches, and cluster headaches. An example of typical patient images comparing a patient without headaches to a patient with recurrent headaches is shown in Figure 2. *By the time of the most recent data collection*, a total of 137 patients had been diagnosed with recurrent headaches compared to the remaining 63, indicating that 15 patients had developed headaches since the initial scans had been taken. Of those 15 patients, 2 were diagnosed with tension headaches, 6 with migraines, and the rest (7) with unspecified headaches, thus, of the 137 total patients now experiencing headaches, 70 (51.1%) were experiencing unspecified headaches, 63 (45.9%) migraines, 6 (4.4%) tension headaches, and 1 (0.7%) cluster headaches. Among the 137 patients who now suffered from headaches, the median NHMR was 0.925 compared to 0.784 for the rest of the 200 patients (63 patients). This difference was significant (*p* < 0.001) (Table 2).

**Table 2:** Mann–Whitney test: comparing the nose-to-heart max ratio of patients with and without headaches during the initial scan and follow-up period.

	At the time of scan	After the scan
Number of patients with headaches	122	137
Number of patients without headaches	78	63
Median NHMR for patients with headaches	0.920	0.925
Median NHMR for patients without headaches	0.818	0.784
*p*-value for NHMR in patients with headaches vs. patients without headaches	0.004^a^	<0.001^a^

NHMR, nose-to-heart max ratio.

^a^ Statistically significant.

Multivariable analysis using gender, diabetes, hypertension, sleep apnea, hyperlipidemia, antihypertensive medication, anti-lipid medication, antihyperglycemic medication, and NHMR as explanatory variables revealed sleep apnea (*p* = <0.001) and NHMR (*p* = 0.004) made significant independent contributions to the patients’ likelihood of having recurrent headaches (Table 3).

**Table 3:** Logistic regression model: clinical predictors of headache diagnosis at the time of initial whole-body blood pool scan.

Characteristic	OR	95% CI	*p*-value
Male	−0.72	−1.8–0.29	0.166
Diabetes	−1.0	−2.9–0.91	0.289
Hypertension	0.45	−1.5–0.58	0.397
Sleep Apnea	1.6	0.70–2.6	<0.001^a^
Hyperlipidemia	0.95	0.04–1.9	0.047
Presence of anti-hypertensive agent	0.35	−0.63–1.4	0.482
Presence of anti-lipid agent	−0.70	−1.7–0.25	0.153
Presence of anti-hyperglycemic agent	0.32	−1.6–2.3	0.747
Nose-to-heart max ratio	2.3	0.80–4.0	0.004^a^

OR, odds ratio; CI, confidence interval.

^a^ Statistically significant.

A multivariable analysis using the same covariates to predict recurrent headaches at the most recent time of data collection identified the same two variables as statistically significant predictors (*p* = <0.001 and *p* = 0.004, respectively, Table 4).

**Table 4:** Logistic regression model: clinical predictors of headache diagnosis during recent data collection.

Characteristic	OR	95% CI	*p*-value
Male	0.42	0.15–1.18	0.103
Diabetes	0.47	0.05–4.74	0.506
Hypertension	0.79	0.25–2.42	0.676
Sleep Apnea	14.2	3.92–92.5	<0.001^a^
Hyperlipidemia	2.23	0.81–6.60	0.132
Presence of anti-hypertensive agent	1.09	0.38–3.17	0.869
Presence of anti-lipid agent	0.68	0.23–1.97	0.473
Presence of anti-hyperglycemic agent	0.82	0.08–8.17	0.859
Nose-to-heart max ratio	12.3	2.34–75.5	0.004^a^

OR, odds ratio; CI, confidence interval.

^a^ Statistically significant.

Among the 122 patients with recurrent headaches at the time of initial imaging, 16 were taking topiramate to treat their headaches. The median NHMR for those patients was 0.815. The other 106 patients with headaches who were not taking topiramate had a median NHMR of 0.933. The *Mann–Whitney test* showed this difference to be significant (U = 572, *P* = 0.033, Table 5). However, *multivariable analysis* using the diagnosis of recurrent headaches at the time of the initial scan, the diagnosis of sleep apnea, the number of headache medications, and each individual medication including topiramate, sumatriptan, amitriptyline, and Fioricet to predict patients’ NHMRs, yielded the results depicted in Table 6 (none of the variables were found to be significant apart from headaches and sleep apnea).

**Table 5:** Mann–Whitney test: comparing the nose-to-heart max ratio of patients taking topiramate for headaches at the time of the initial scan to that of patients with headaches who were not taking topiramate.

	At the time of initial scan
Number of patients with headaches taking topiramate	16
Number of patients with headaches not taking topiramate	106
Median NHMR for patients with headaches taking topiramate	0.815
Median NHMR for patients with headaches not taking topiramate	0.933
*p*-value for NHMR in patients with headaches taking topiramate vs. patients with headaches not taking topiramate	0.033^a^

^a^ Statistically significant; NHMR, nose-to-heart max ratio.

**Table 6:** Linear model: headache diagnosis and medications vs. nose-to-heart max ratio.

Characteristic	OR	95% CI	*p*-value
Sleep apnea	0.11	0.03 to 0.18	0.006^a^
Headache diagnosis	0.10	0.03 to 0.17	0.007^a^
Number of headache medications	−0.03	−0.17 to 0.10	0.615
Topiramate	−0.06	−0.27 to 0.16	0.604
Amitriptyline	0.11	−0.01 to 0.22	0.072
Sumatriptan	0.05	−0.13 to 0.23	0.580
Fioricet	0.02	−0.20 to 0.24	0.844

OR, odds ratio; CI, confidence interval.

^a^ Statistically significant.

In summary, NHMR significantly correlated with the presence of recurrent headaches as well as with headaches that began 3–6 years later. Additionally, while a previous study suggested that NHMR correlated with metabolic syndrome (19), which is associated with headaches, multivariable analysis to predict headaches identified an independent and significant contribution to risk made by NHMR alone, even after considering other components of metabolic syndrome. Finally, while multivariable analysis investigating the contributions made by specific headache medications to NHMR failed to produce statistically significant results for topiramate (*P* = 0.604), the difference in median NHMR between patients taking topiramate for headaches and patients with headaches but who were not taking the drug suggests that topiramate may reduce NHMR.

## 4 Discussion

Approximately 90% of individuals will experience headache disorders at some point in their lives (24). Migraine, a subtype of headaches, ranks among the world's most prevalent neurological disorders, affecting an estimated 1.1 billion individuals globally (25). The rising prevalence of recurrent headaches highlight their significant impact on quality of life, necessitating research on their etiologies and more effective treatment strategies. In our retrospective study of 200 patients, we explored the associations of specific signs and symptoms in patients with recurrent headaches and their nose-to-heart max ratio (NHMR) as observed on whole-body blood pool scans.

Our findings indicate a significant correlation between elevated nasal blood pool ratios and recurrent headaches. The significantly increased nasal activity seen on whole-body blood pool scans can be attributed to increased nasal turbinate blood volumes. The dilation of nasal turbinates is regulated by the autonomic nervous system, where enhanced *parasympathetic* activity leads to dilation, and heightened *sympathetic* activity results in constriction (26). The increased nasal blood pool ratios suggest that recurrent headaches may be a result of increased parasympathetic activity resulting in dilation of nasal turbinates and resultant blood pooling. The involvement of parasympathetic innervation in the meninges is a crucial aspect of headache pathophysiology. Neurotransmitters like acetylcholine, vasoactive intestinal peptide, and pituitary adenylate-cyclase-activating polypeptide, released from parasympathetic nerve fibers, significantly contribute to the process of dural pain sensation (27).

**Figure 3:**
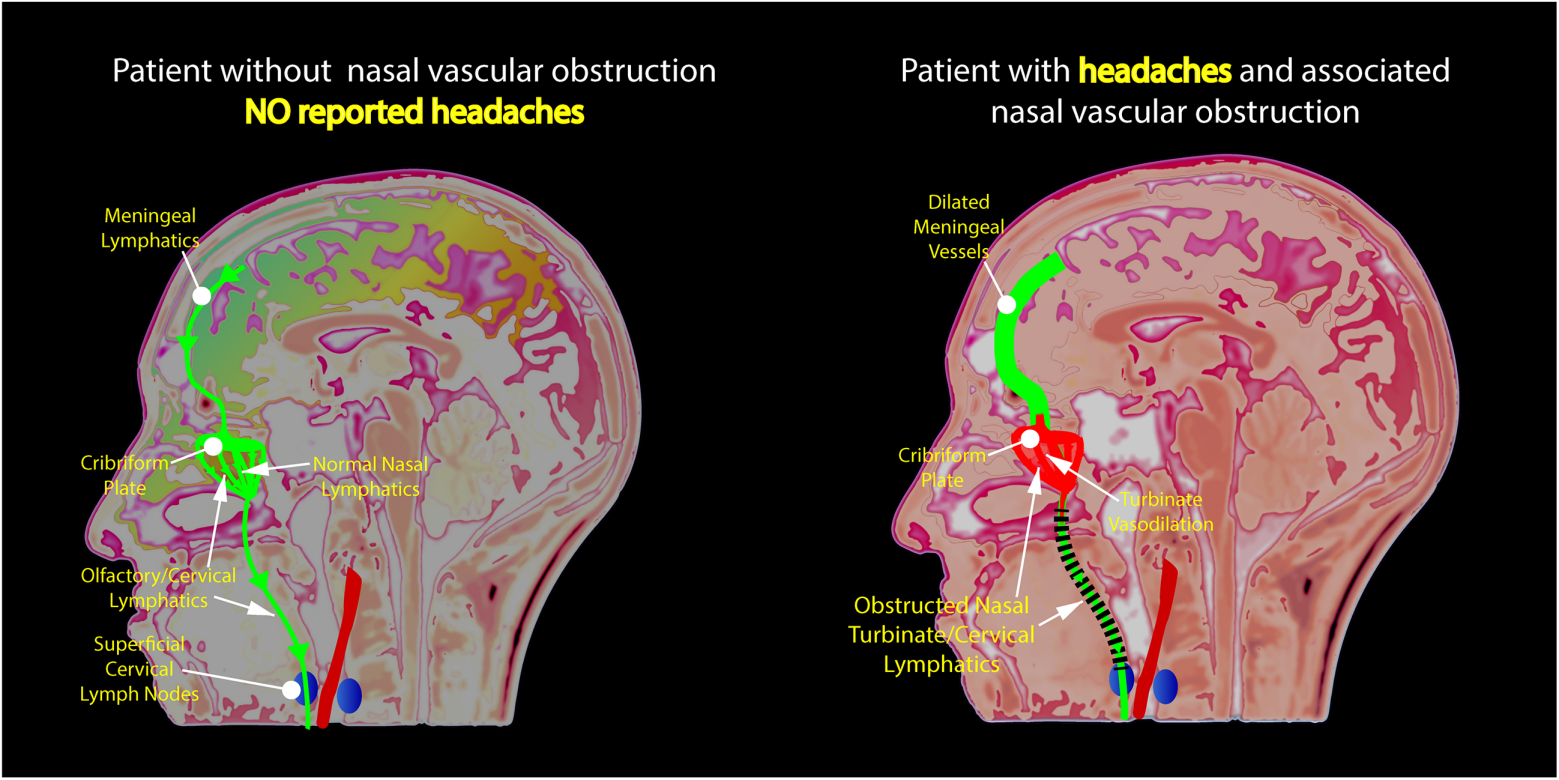
Illustration of the authors’ novel hypothesis for the etiology of nasal congestion and chronic headaches revealing obstruction of lymphatic flow due to the dilation of nasal turbinate vessels.

In recent years, the nasal lymphatic route of cerebrospinal fluid (CSF) outflow has gained increasing attention from researchers and practitioners alike. The nasal lymphatic system is integral to fluid balance and waste removal from the cranial space (14). Nasal submucosal lymphatic vessels, directly linked to the subarachnoid space, facilitate intracranial CSF drainage, establishing a conduit between nasal lymphatics, the olfactory system, and the brain (16). Studies have shown that the human nasal turbinates are integrated into the CSF clearance network. Enhanced vasodilation and blood pooling in the nasal turbinates may potentially disrupt the clearance of brain waste proteins which travel from the CSF through the cribriform plate and into the lymphatics of the nasal turbinates (15). Figure 3 shows a proposed mechanism of how nasal turbinate vasodilation may contribute to nasal lymphatic obstruction and lead to the development of headaches. Anomalies in CSF clearance have been identified in Alzheimer's disease, providing evidence for a physiological CSF pathway exiting from the cribriform plate, through the nasal turbinates, and into the cervical lymphatics (28, 29).

The glymphatic network serves as a fluid-clearance system, clearing waste from brain parenchyma into perivascular spaces, which then empty into the dural lymphatic network that envelops the brain, and from there, it proceeds to lymphatic vessels that follow the paths of cranial nerves and major blood vessels (30). Studies indicate that the perivascular glymphatic pathway, driven by aquaporin 4 channel-dependent bulk flow, plays a substantial role in the removal of interstitial fluid solutes from the brain's parenchyma and toxic metabolites such as β-amyloid (31).

Our results align with the emerging research on the glymphatic system's role in neurovascular and neuroinflammatory pathologies (30–33). Recent research demonstrates that cortical spreading depression, identified as the physiological basis of migraine aura, constricts the perivascular space, thereby hindering glymphatic circulation (34). This insight suggests that the glymphatic pathway, integral to clearing metabolic waste from the brain, may be compromised and lead to disruption of cortical and endothelial functions, potentially contributing to the pathogenesis of headaches. Therefore, recurrent headaches may result from decreased clearance of brain CSF through the dilated, blood-filled, nasal turbinates (35, 36). The increased nasal blood pooling seen in our whole-body blood pool scans in patients suffering from recurrent headaches suggests a significant role of disrupted perivascular clearance pathways in the neuroinflammation-involved headache pathology (36, 37).

Nicholson et al. have described intracranial hypertension as glymphedema of the brain (38). Glymphatic disruption has been described in another headache-associated entity, idiopathic intracranial hypertension (IIH) in which studies have shown important links between migraine, IIH, and obesity (38, 39). A recently published study using transcranial doppler of the intracranial arteries to evaluate patients with IIH, migraine headaches, and healthy controls, found mean flow velocities (MFV) of the middle cerebral artery were significantly correlated with CSF pressures and were highest in patients with IIH; however, patients with migraine headaches also had significantly higher MFVs as compared to controls (*p* < 0.001) (40). These studies provide evidence of elevated CSF pressures occurring in patients with migraine headaches. Similar findings were found with resistivity indices which were also significantly higher in IIH and migraine patients when compared to controls (*p* < 0.001). Resistivity indices are also positively correlated with CSF pressures. These findings show a continuum of CSF pressure findings between IIH, migraines, and controls. Nasal lymphatic obstruction could be a common pathway linking these entities.

The authors found that patients who are using topiramate had statistically significant *decreased* nasal blood pooling on whole-body blood pool scans. However, this association was found to be statistically insignificant for recurrent headaches, likely due to too few patients taking the drug (*n* = 16).

We acknowledge several limitations in our study. The retrospective design and reliance on previously collected data may introduce biases and limit the ability to establish causality. The problem of poorly defined headache types in this retrospective study limits the interpretation of our results; however, a future prospective study correlating the images with headache type, duration, and severity would be valuable and could improve the significance of the NHMR correlations. The demographic skew towards females, reflective of rheumatoid arthritis prevalence, may affect the generalizability of our findings to other populations. Additionally, the exclusion of variables in the statistical analysis due to incomplete data sets underscores the need for comprehensive prospective studies to further validate our findings. Finally, the lack of clarity in the patients’ charts concerning which form of headache roughly half of the patients in the study were suffering from precluded a more detailed analysis of NHMR by subtype, clouding the generalizability of our findings.

Prior to adopting the max pixel ratios used in these studies, we experienced challenges relating to the performance of image analysis, notably, the variability and potential subjectivity introduced by delineating a hand-drawn region of interest around the nasal area, as the pixel counts can differ depending on how the region of interest is drawn. To eliminate any potential subjectivity, our statistical analysis concentrated on the maximum pixel activity which is a measure that remains consistent across varying region sizes. The use of maximum pixel counts in our study mirrors the approach of using peak intensity values in MRI diffusion imaging, where peak intensity values standardize measurements across different anatomical regions, in PET cancer imaging using max standard uptake values (MaxSUV), and in echocardiography, where peak velocity measurements across cardiac valves ensure uniform assessments despite anatomical differences. Both approaches effectively minimize interpretation variations due to diverse region sizes.

The strength of our study lies in the capability of whole-body nuclear imaging to distinctly depict the activity of the nasal blood pool in relation to other great vessels as well as the heart. The ability to provide whole-body imaging with ratio comparisons is a unique capability of nuclear imaging, a feature unattainable with alternative imaging techniques like CT or MRI. Another notable advantage is that we used quantitative data, permitting the statistical analysis of nasal blood pool activity ratios across a large patient cohort. If the findings in our study are substantiated, whole-body blood pool imaging may serve as a diagnostic tool to identify patients with the highest risk of developing headache disorders. Utilizing the nose-to-heart max ratio as a predictive marker, nuclear imaging scans may allow for early detection and intervention of recurrent headache disorders (41, 42).

## 5 Conclusion

Headache disorders are a rising public health challenge. The increased nasal activity observed on blood pool scans is a significant and independent predictor for headaches, indicating a potential underlying pathophysiological mechanism. This research also points to a possible new diagnostic use of the whole-body blood pool scan to assess patients for nasal turbinate vasodilation as a cause of recurrent headaches. If verification of the association of nasal turbinate vasodilation with headaches is found in future prospective studies, these findings would have important implications for both diagnosing the risk of recurrent headaches and developing novel headache treatments with nasal turbinates as a new therapeutic target.

## Data Availability

The datasets presented in this study can be found in online repositories. The names of the repository/repositories and accession number(s) can be found below: Phillips, William, 2024, "Correlation of nose max to heart max ratios with metabolic syndrome, sleep apnea and headache", doi: 10.18738/T8/SN3KS9, Texas Data Repository.
